# Knowledge and Practice of Precautions and Prevention of COVID-19 Among Adolescents in Umuahia, Nigeria: A Cross-Sectional Study

**DOI:** 10.7759/cureus.64984

**Published:** 2024-07-20

**Authors:** Pamela Chidinma Orunta, Chinomso Obianuju Ndu, Chimaobi Ezekiel Ijioma, Oboseh J Ogedegbe, Funmilola Abosede Ogundipe, Chioma P Eze-Njoku

**Affiliations:** 1 Pediatrics, Federal Medical Centre, Umuahia, NGA; 2 Family Medicine, North Texas Family Practice, Mesquite, USA; 3 Pediatrics, Abia State Specialist Hospital and Diagnostics Center, Umuahia, NGA; 4 Internal Medicine, Lifeway Medical Centre, Abuja, NGA; 5 Family Medicine, Saskatchewan Health Authority, Saskatoon, CAN; 6 Pediatrics, Atrium Health Navicent, Macon, USA

**Keywords:** precaution, prevention, practice, knowledge, covid-19, adolescent

## Abstract

Background

The coronavirus disease 2019 (COVID-19) pandemic has posed significant challenges worldwide, particularly in developing countries with limited healthcare resources. Among the various demographics, adolescents represent a crucial group in understanding and curbing the spread of the virus.

Aim

This research aims to assess the level of knowledge and practice of COVID-19 prevention measures among adolescents in a developing country.

Methodology

This study's descriptive cross-sectional study design was appropriate for capturing a snapshot of adolescents' understanding of COVID-19 in Umuahia, Abia State, Nigeria, a developing country. The study's participants were secondary school students in Umuahia's urban and rural secondary schools, aged 10-19. Fisher's formula was used to calculate the sample size. A multistage systematic sampling method was used to select 424 participants. Data were gathered using a self-administered questionnaire. The Statistical Package for the Social Sciences (SPSS) software version 25 (IBM SPSS Statistics, Armonk, NY) was used to handle and evaluate the data that had been obtained.

Results

All the respondents were aware of COVID-19, primarily informed through social media and television (TV). It is concerning that less than half correctly identify the disease as caused by a virus (46.9%), with some falsely attributing it to bacteria (31.1%) or fungi (15.6%). While the majority of respondents knew about the existence of COVID-19 vaccines, only 63.4% were aware that there was no definitive cure for the disease. The most worrying finding was the limited awareness and practice of recommended precautions to prevent the spread of COVID-19. Handwashing with soap and water, social distancing, and using hand sanitizers were the most frequently recognized precautions. However, even these were poorly practiced, with less than 30% of the respondents following them regularly. It was also noted that misconceptions about precautions exist, including unusual practices such as regularly drinking hot water or eating bitter kola/pepperfruit. Also, the most prominent reason for refusal among the respondents for COVID-19 vaccines was parental refusal, accounting for 57.5% of negative responses.

Conclusion

The study's findings underscore the urgent need for tailored, accessible, and effective health education strategies to improve adolescents' understanding and adoption of COVID-19 preventive measures in the region.

## Introduction

The novel coronavirus disease 2019 (COVID-19) pandemic, caused by the severe acute respiratory syndrome coronavirus 2 (SARS-CoV-2), has posed an unparalleled global public health crisis. Originating from Wuhan, China, in late 2019 [1], the disease has since spread to over 200 countries and territories, affecting millions worldwide. The implications of the pandemic extend beyond health, disrupting various aspects of human life, including socioeconomic and educational structures [2].

Adolescents, aged 10-19, are among the affected populations [3]. This population segment possesses a unique set of vulnerabilities, largely due to their developmental stage. The role of adolescents in the pandemic's trajectory is significant, owing to their high mobility and substantial social interactions. Although generally experiencing milder COVID-19 disease symptoms than adults, adolescents can contribute to virus transmission [4].

In Nigeria, Abia State has faced its share of the COVID-19 pandemic challenge, with Umuahia, the capital city, being a significant hotspot. An understanding of the knowledge and practices regarding COVID-19 precautions and prevention among adolescents in this region is critical to mitigating the disease's spread effectively.

Previous studies have delved into various aspects of COVID-19 knowledge and preventative practices. However, many of these focus on healthcare workers or the general adult population [5,6]. Understanding the depth of knowledge, attitudes, and practices among adolescents toward COVID-19 can help tailor public health interventions and education campaigns targeting this demographic [7].

Furthermore, the importance of community-based health education and promotion interventions has been emphasized in the wake of this pandemic [8]. There is a need for more localized studies that can provide an in-depth understanding of the knowledge and practices of specific communities, which can then inform tailored and effective interventions. This study focuses on the knowledge and practice of precautions and prevention of COVID-19 among adolescents in Umuahia, Abia State, Nigeria.

## Materials and methods

Study area

The state capital of Abia is Umuahia, which is located in southeast Nigeria. It has 359,230 residents (2006 Nigerian census). Umuahia is divided into two local government areas (LGAs): Umuahia North and Umuahia South. Five clans make up these LGAs collectively: Umuopara, Ibeku, Olokoro, Ubakala, and Ohuhu. With a total enrollment of 48,267 people, Umuahia has 86 secondary schools, 56 of which are in Umuahia North and 30 in Umuahia South LGAs. Most of the inhabitants are traders, students, and civil servants.

Research design

A cross-sectional study design was appropriate for capturing a snapshot of adolescents' knowledge and practice of precautions and prevention of COVID-19 in Umuahia, Abia State, Nigeria. The study's participants were secondary school students in Umuahia's urban and rural schools.

Inclusion criteria

Inclusion criteria include all adolescents aged 10-19 who are registered in the secondary schools in Umuahia and who gave consent or obtained parental consent.

Exclusion criteria

Exclusion criteria include students and parents who did not give consent/refused to participate in the study, students below 10 years of age or above 19 years, and adolescents who are not registered in the schools.

Sample size determination

The sample size was determined using Fisher's formula [9]: n˳= Z² p q/e²; n˳= the desired sample size (when the population is greater than 10,000); Z = standard normal deviation set at 1.96, which corresponds to the 95% confidence level; p = population in the target population estimated to have good knowledge of COVID-19 (it will be set at 50% (because there is no previously established prevalence)); q = 1.0 - p; e = degree of precision required (0.05); n˳= (1.96)² (0.50) (0.50)/ (0.05)²; and n˳= 384. An attrition rate of 10% was added, giving a total of 384 + 38 = 422. An attrition rate of 10% was added to ensure the validity and reliability of the study results by accounting for participants who would possibly drop out.

Sampling technique

A multistage systematic sampling method was used. In the first stage, the 86 government-approved schools in Umuahia were stratified based on gender (three male-only, five female-only, and 78 mixed schools), proprietorship status (32 public and 54 private schools), and location (17 urban and 69 rural schools). Selections were made based on the ratio of the various schools in each stratum. The male-only (n = 3), female-only (n = 5), and mixed schools (n = 78) were first stratified. One school each was randomly chosen from the male- and female-only schools. The remaining 78 schools were further stratified into public (n = 27) and private (n = 51) schools, from which one public and two private schools were randomly chosen, respectively, based on the ratio of their proportion. The last stratum was based on location. From the 17 rural and 69 urban schools in this stratum, one rural and four urban schools were randomly selected also based on the ratio of their proportion. A total of 10 schools were chosen: one male-only, one female-only, one public, two private, one rural, and four urban schools. These selections were done strictly by simple random selection after blinding (each school was written on a piece of paper, folded, and put in a container from which the selections were made). A school that has been previously chosen from a particular stratum was not repeated in the next stratum during selection.

In the second stage, the number of students to be administered questionnaires in each school was worked out using the number of students in each school as a proportion of the total number of students in Umuahia as follows: (number of students in the school/total number of students in Umuahia) × minimum sample size for the study. A sampling frame with blocks of four was used to select students from each class in the schools surveyed. All the students in each class were grouped into four, and the first student from each frame was selected as a respondent until the required number was obtained.

Assessment instrument

A self-administered online questionnaire was used to assess respondents' knowledge and practice of precautions and prevention of COVID-19. The questionnaire covered the demographic information of the respondents, knowledge of the cause and cures for COVID-19, knowledge of precautions for COVID-19 as well as the ones they follow, source of information on COVID-19 vaccines, choice of taking, and reasons for refusal if indicated.

Ethical consideration

Participants were fully informed about the study's purpose, procedures, and their rights, including the right to withdraw at any time without any consequences. Informed consent was obtained electronically, ensuring that participants were aware of how their data would be used and protected. Ethical approval for the study was obtained from the Ethics Committee for Public Health Research, Department of Public Health and Disease Control, Abia State Ministry of Health, Umuahia, Nigeria, with approval number ASMH/EC/23/014. Personal identifiers such as names, addresses, or any other identifying information were not collected during the study. Participants were assigned numbers to ensure anonymity.

Data analysis

The Statistical Package for the Social Sciences (SPSS) software version 25 (IBM SPSS Statistics, Armonk, NY) was used to handle and evaluate the data that had been obtained. For each categorical variable, descriptive statistics (frequencies and percentages) were calculated. Chi-square tests were used in a bivariate study to find correlations between independent and dependent variables.

The identification of predictors of knowledge and practice on the precautions and prevention of COVID-19 was accomplished using multivariate logistic regression analysis. A P-value of 0.05 was chosen as the cutoff for statistical significance. Also, a regression analysis was carried out to assess the level of knowledge and practice on the precautions and prevention of COVID-19.

## Results

The results for the demographic information of the respondents in this survey are presented in Table 1. Out of 424 respondents, about 51% were male and 49% were female. The majority were between the ages of 13 and 15 (36.1%), and the fewest were 19 years old (8.3%). Most respondents were Christian (76.7%), while a smaller proportion were Muslim (20.8%), and the rest followed traditional beliefs. A significant number of respondents did not know their father's level of education (22.2%), and most mothers had tertiary education (39.6%). Every respondent was aware of COVID-19, and the majority learned about it through social media (46.9%) or television (TV) (31.1%). The majority also knew that COVID-19 is caused by a virus (83.5%).

**Table 1:** Demographic characteristics of the respondents COVID-19: coronavirus disease 2019, TV: television

Characteristic	Subgroups	N = 424			Percentage (%)
Gender	Male	215			50.7
	Female	209			49.3
Age	10-12	122			28.8
	13-15	153			36.1
	16-18	114			26.9
	19	35			8.3
Religion	Christianity	325			76.7
	Islam	88			20.8
	Traditional	11			2.6
Father's level of education	I don't know	94			22.2
	No education	59		13.9	
	Primary	85		20	
	Secondary	85		20	
	Tertiary	101		23.82	
Mother's level of education	I don't know	89		21	
	No education	43	10.1		
	Primary	36	8.5		
	Secondary	88	20.8		
	Tertiary	168	39.6		
Awareness of COVID-19	Yes	424	100		
Sources of awareness of COVID-19	Social media	199	46.9		
	TV	132	31.1		
	Radio	66	15.6		
	Family	1	0.2		
	School	6	1.4		
	Church	4	0.9		
	Mosque	11	2.6		
	Newspapers	1	0.2		
	Friends	4	0.9		

The responses based on the knowledge of the respondents about the causes of COVID-19 are presented in Table 2. Less than half correctly identify the disease as caused by a virus (46.9%), with some falsely attributing it to bacteria (31.1%) or fungi (15.6%).

**Table 2:** Knowledge of the respondents about the causes of COVID-19 COVID-19: coronavirus disease 2019

Causes of COVID-19	Yes	No	I don't know
Virus	199 (46.9%)	224 (52.8%)	1 (0.2%)
Bacteria	132 (31.1%)	291 (68.6%)	1 (0.2%)
Fungi	66 (15.6%)	358 (84.4)	1 (0.2%)

Regarding the cure, as seen in Table 3, 63.4% believe that the COVID-19 infection has a cure, and 84.2% are aware of the existence of vaccines and immunizations. Also, 73.6% correctly view COVID-19 as a problem in Nigeria and worldwide.

**Table 3:** Knowledge of the respondents about the cure of COVID-19 COVID-19: coronavirus disease 2019

Item	Yes	No	I don't know
Does COVID-19 infection have a permanent cure?	269 (63.4%)	57 (13.4%)	98 (23.1%)
Are there vaccines/immunizations against COVID-19?	357 (84.2%)	23 (5.4%)	44 (10.4%)
Is COVID-19 a problem in Nigeria and the whole world?	312 (73.6%)	43 (10.1%)	69 (16.3%)

Table 4 details precautions that respondents believe can mitigate the spread of COVID-19. The measures are diverse, ranging from scientifically supported ones such as "handwashing with soap and water" (8.5% yes) and "social distancing" (6.1% yes) to more dubious methods such as "eating bitter kola/pepperfruit." It is alarming to see low acknowledgment of key preventive measures such as wearing masks and vaccination.

**Table 4:** Knowledge of the respondents on the precautions to reduce COVID-19 COVID-19: coronavirus disease 2019

Precaution	Yes	No
Nil	1 (0.2%)	423 (99.8%)
Avoiding crowded places	5 (1.2%)	419 (98.8%)
Avoiding crowded places; special prayers; regularly drinking hot water	1 (0.2%)	423 (99.8%)
COVID-19 vaccination	5 (1.2%)	419 (98.8%)
COVID-19 vaccination; eating bitter kola/pepperfruit	1 (0.2%)	423 (99.8%)
COVID-19 vaccination; regularly drinking hot water	2 (0.5%)	422 (99.5%)
Hand washing with soap and water	36 (8.5%)	388 (91.5%)
Hand washing with soap and water; avoiding crowded places	10 (2.4%)	414 (97.6)
Hand washing with soap and water; avoiding crowded places; bathing with salt water	4 (0.9%)	420 (99.1%)
Hand washing with soap and water; avoiding crowded places; COVID-19 vaccination	9 (2.1%)	415 (97.9%)
Hand washing with soap and water; avoiding crowded places; regular use of hand sanitizer	2 (0.5%)	422 (99.5)
Hand washing with soap and water; avoiding crowded places; special prayers	1 (0.2%)	423 (99.8%)
Hand washing with soap and water; bathing with salt water; drinking concoctions like garlic, turmeric, and lime	1 (0.2%)	423 (99.8%)
Hand washing with soap and water; bathing with salt water; eating bitter kola/pepperfruit	1 (0.2%)	423 (99.8%)
Hand washing with soap and water; bathing with salt water	1 (0.2%)	423 (99.8%)
Hand washing with soap and water; COVID-19 vaccination	4 (0.9%)	420 (99.1%)
Hand washing with soap and water; COVID-19 vaccination; bathing with salt water	5 (1.2%)	419 (98.8%)
Hand washing with soap and water; COVID-19 vaccination; eating bitter kola/pepperfruit	5 (1.2%)	419 (98.8%)
Hand washing with soap and water; COVID-19 vaccination; regularly drinking hot water	5 (1.2%)	419 (98.8%)
Hand washing with soap and water; COVID-19 vaccination; special prayers	7 (1.7%)	417 (98.3%)
Hand washing with soap and water; drinking concoctions like garlic, turmeric, and lime	2 (0.5%)	422 (99.5)
Hand washing with soap and water; regular use of hand sanitizer	11 (2.6%)	413 (97.4%)
Hand washing with soap and water; regular use of hand sanitizer; regularly drinking hot water	51 (12%)	373 (88%)
Hand washing with soap and water; regularly drinking hot water	11 (2.6%)	413 (97.4%)
Hand washing with soap and water; social distancing	20 (4.7%)	404 (95.3%)
Hand washing with soap and water; social distancing; avoiding crowded places	27 (6.4%)	397 (93.6%)
Hand washing with soap and water; social distancing; bathing with salt water	1 (0.2%)	423 (99.8%)
Hand washing with soap and water; social distancing; COVID-19 vaccination	13 (3.1%)	411 (96.9%)
Hand washing with soap and water; social distancing; regular use of hand sanitizer	116 (27.4%)	308 (72.6%)
Hand washing with soap and water; social distancing; regularly drinking hot water	1 (0.2%)	423 (99.8%)
Hand washing with soap and water; social distancing; special prayers	1 (0.2%)	423 (99.8%)
Hand washing with soap and water; special prayers	1 (0.2%)	423 (99.8%)
Hand washing with soap and water; special prayers; bathing with salt water	2 (0.5%)	422 (99.5%)
Hand washing with soap and water; special prayers; drinking concoctions like garlic, turmeric, and lime	1 (0.2%)	423 (99.8%)
Hand washing with soap and water; special prayers; eating bitter kola/pepperfruit	2 (0.5%)	422 (99.5%)
Hand washing with soap and water; use of mask	1 (0.2%)	423 (99.8%)
Regular use of hand sanitizer	3 (0.7%)	421 (99.3%)
Regular use of hand sanitizer; COVID-19 vaccination	2 (0.5%)	422 (99.5%)
Regularly drinking hot water	1 (0.2%)	423 (99.8%)
Social distancing	26 (6.1%)	398 (93.9%)
Social distancing; avoiding crowded places	2 (0.5%)	422 (99.5)
Social distancing; avoiding crowded places; COVID-19 vaccination	1 (0.2%)	423 (99.8%)
Social distancing; avoiding crowded places; regularly drinking hot water	1 (0.2%)	423 (99.8%)
Social distancing; avoiding crowded places; special prayers	2 (0.5%)	422 (99.5%)
Social distancing; bathing with salt water; regularly drinking	3 (0.7%)	421 (99.3%)
Social distancing; COVID-19 vaccination	1 (0.2%)	423 (99.8%)
Social distancing; COVID-19 vaccination; bathing with salt water	2 (0.5%)	422 (99.5%)
Social distancing; COVID-19 vaccination; special prayers; regular use of hand sanitizer	1 (0.2%)	423 (99.8%)
Social distancing; regular use of hand sanitizer	2 (0.5%)	422 (99.5%)
Social distancing; regular use of hand sanitizer; COVID-19 vaccination	3 (0.7%)	421 (99.3%)
Social distancing; regular use of hand sanitizer; eating bitter kola/pepperfruit	1 (0.2%)	423 (99.8%)
Social distancing; regularly drinking hot water	3 (0.7%)	421 (99.3%)
Social distancing; special prayers	1 (0.2%)	423 (99.8%)
Special prayers	2 (0.5%)	422 (99.5%)

Table 5 provides insight into the actions respondents are actually taking to protect themselves from COVID-19. The most common precaution is "handwashing with soap and water," with 12.5% of respondents saying they follow this. Other notable precautions, such as "avoiding crowded places" and receiving the "COVID-19 vaccination," have low rates of compliance. There is a discrepancy between knowledge and practice, which may contribute to the ongoing spread of the virus.

**Table 5:** Precautions that respondents follow COVID-19: coronavirus disease 2019

Precaution	Yes	No
Nil	5 (1.2%)	419 (98.8%)
Avoiding crowded places	3 (0.7%)	421 (99.3%)
Avoiding crowded places; COVID-19 vaccination	1 (0.2%)	423 (99.8%)
COVID-19 vaccination	1 (0.2%)	423 (99.8%)
COVID-19 vaccination; eating bitter kola/pepperfruit; drinking concoctions like garlic, turmeric, and lime	1 (0.2%)	423 (99.8%)
COVID-19 vaccination; regularly drinking hot water	1 (0.2%)	423 (99.8%)
Eating bitter kola/pepperfruit	1 (0.2%)	423 (99.8%)
Hand washing with soap and water	53 (12.5%)	371 (87.5%)
Hand washing with soap and water; avoiding crowded places	12 (2.8%)	412 (97.2%)
Hand washing with soap and water; avoiding crowded places; bathing with salt water	1 (0.2%)	423 (99.8%)
Hand washing with soap and water; avoiding crowded places; COVID-19 vaccination	3 (0.7%)	421 (99.3%)
Hand washing with soap and water; avoiding crowded places; eating bitter kola/pepperfruit	1 (0.2%)	423 (99.8%)
Hand washing with soap and water; avoiding crowded places; regularly drinking hot water	3 (0.7%)	421 (99.3%)
Hand washing with soap and water; avoiding crowded places; special prayers	6 (1.4%)	418 (98.6%)
Hand washing with soap and water; bathing with salt water	1 (0.2%)	423 (99.8%)
Hand washing with soap and water; bathing with salt water; eating bitter kola/pepperfruit	1 (0.2%)	423 (99.8%)
Hand washing with soap and water; bathing with salt water; regularly drinking hot water	2 (0.5%)	422 (99.5%)
Hand washing with soap and water; COVID-19 vaccination	2 (0.5%)	422 (99.5%)
Hand washing with soap and water; COVID-19 vaccination; bathing with salt water	2 (0.5%)	422 (99.5%)
Hand washing with soap and water; COVID-19 vaccination; eating bitter kola/pepperfruit	1 (0.2%)	423 (99.8%)
Hand washing with soap and water; COVID-19 vaccination; regularly drinking hot water	12 (2.8%)	412 (97.2%)
Hand washing with soap and water; COVID-19 vaccination; special prayers	4 (0.9%)	420 (99.1%)
Hand washing with soap and water; face/nose mask	1 (0.2%)	423 (99.8%)
Hand washing with soap and water; regular use of hand sanitizer	13 (3.1%)	411 (96.9%)
Hand washing with soap and water; regular use of hand sanitizer; social distancing	44 (10.4%)	380 (89.6%)
Hand washing with soap and water; regularly drinking hot water	7 (1.7%)	417 (98.3%)
Hand washing with soap and water; regularly drinking hot water; drinking concoctions like garlic, turmeric, and lime	1 (0.2%)	423 (99.8%)
Hand washing with soap and water; social distancing	31 (7.3%)	393 (92.7%)
Hand washing with soap and water; social distancing; avoiding crowded places	21 (5%)	403 (95%)
Hand washing with soap and water; social distancing; bathing with salt water	1 (0.2%)	423 (99.8%)
Hand washing with soap and water; social distancing; COVID-19 vaccination	8 (1.9%)	416 (98.1%)
Hand washing with soap and water; social distancing; eating bitter kola/pepperfruit	1 (0.2%)	423 (99.8%)
Hand washing with soap and water; social distancing; regular use of hand sanitizer	81 (19.1%)	343 (80.9%)
Hand washing with soap and water; social distancing; regularly drinking hot water	5 (1.2%)	419 (98.8%)
Hand washing with soap and water; social distancing; special prayers	3 (0.7%)	421 (99.3%)
Hand washing with soap and water; special prayers	4 (0.9%)	420 (99.1%)
Hand washing with soap and water; special prayers; drinking concoctions like garlic, turmeric, and lime	2 (0.5%)	422 (99.5%)
Hand washing with soap and water; special prayers; regularly drinking hot water	2 (0.5%)	422 (99.5%)
Hand washing with soap and water; face/nose mask	1 (0.2%)	423 (99.8%)
Regular use of hand sanitizer	3 (0.7%)	421 (99.3%)
Regular use of hand sanitizer; avoiding crowded places; regularly drinking hot water	1 (0.2%)	423 (99.8%)
Regular use of hand sanitizer; COVID-19 vaccination; special prayers	1 (0.2%)	423 (99.8%)
Regular use of hand sanitizer; drinking concoctions like garlic, turmeric, and lime	1 (0.2%)	423 (99.8%)
Regular use of hand sanitizer; eating bitter kola/pepperfruit	1 (0.2%)	423 (99.8%)
Regular use of hand sanitizer; special prayers; regularly drinking hot water	1 (0.2%)	423 (99.8%)
Regularly drinking hot water	1 (0.2%)	423 (99.8%)
Social distancing	27 (6.4%)	397 (93.6%)
Social distancing; avoiding crowded places	14 (3.3%)	410 (96.7%)
Social distancing; avoiding crowded places; COVID-19 vaccination	1 (0.2%)	423 (99.8%)
Social distancing; avoiding crowded places; special prayers	2 (0.5%)	422 (99.5%)
Social distancing; bathing with salt water; regularly drinking hot water	3 (0.7%)	421 (99.3%)
Social distancing; COVID-19 vaccination	1 (0.2%)	423 (99.8%)
Social distancing; COVID-19 vaccination; eating bitter kola/pepperfruit	2 (0.5%)	422 (99.5%)
Social distancing; COVID-19 vaccination; regularly drinking hot water	2 (0.5%)	422 (99.5%)
Social distancing; COVID-19 vaccination; special prayers; eating bitter kola/pepperfruit	1 (0.2%)	423 (99.8%)
Social distancing; regular use of hand sanitizer	2 (0.5%)	422 (99.5%)
Social distancing; regular use of hand sanitizer; avoiding crowded places	5 (1.2%)	419 (98.8%)
Social distancing; regular use of hand sanitizer; COVID-19 vaccination	3 (0.7%)	421 (99.3%)
Social distancing; regular use of hand sanitizer; special prayers	2 (0.5%)	422 (99.5%)
Social distancing; regularly drinking hot water	3 (0.7%)	421 (99.3%)
Social distancing; special prayers	1 (0.2%)	423 (99.8%)
Special prayers	1 (0.2%)	423 (99.8%)
Special prayers; bathing with salt water; regularly drinking hot water	1 (0.2%)	423 (99.8%)
Special prayers; eating bitter kola/pepperfruit	1 (0.2%)	423 (99.8%)

The responses of the participants on the different sources of COVID-19 vaccines are presented in Table 6. The most common source is television (19.8%), followed by radio (10.6%) and social media (8.3%).

**Table 6:** Knowledge of the respondents on the sources of the COVID-19 vaccines COVID-19: coronavirus disease 2019, TV: television

Item	Yes	No
Nil	9 (2.1%)	415 (97.9%)
Church	3 (0.7%)	421 (99.3%)
Church; school teacher; friends; market	1 (0.2%)	423 (99.8%)
Family member	6 (1.4%)	418 (98.6%)
Friends	9 (2.1%)	415 (97.9%)
Market	1 (0.2%)	423 (99.8%)
Mosque	7 (1.7%)	417 (98.3%)
Mosque; family member	1 (0.2%)	423 (99.8%)
Mosque; market	1 (0.2%)	423 (99.8%)
Not sure	14 (3.3%)	410 (96.7%)
Questionnaire personnel	1 (0.2%)	423 (99.8%)
Radio	45 (10.6%)	375 (89.4%)
Radio; mosque	1 (0.2%)	423 (99.8%)
Radio; church	3 (0.7%)	421 (99.3%)
Radio; church; friends	1 (0.2%)	423 (99.8%)
Radio; family member	6 (1.4%)	418 (98.6%)
Radio; family member; newspaper	1 (0.2%)	423 (99.8%)
Radio; friends	2 (0.5%)	422 (99.5%)
Radio; mosque; school teacher	1 (0.2%)	423 (99.8%)
Radio; newspaper	1 (0.2%)	423 (99.8%)
Radio; school teacher	2 (0.5%)	422 (99.5%)
Random places	1 (0.2%)	423 (99.8%)
School teacher	3 (0.7%)	421 (99.3%)
School teacher; family member	3 (0.7%)	421 (99.3%)
School teacher; friends; family member	1 (0.2%)	423 (99.8%)
Social media (Facebook, Instagram, Twitter, WhatsApp)	35 (8.3%)	389 (91.7%)
Social media (Facebook, Instagram, Twitter, WhatsApp); church	6 (1.4%)	418 (98.6%)
Social media (Facebook, Instagram, Twitter, WhatsApp); family	5 (1.2%)	419 (98.8%)
Social media (Facebook, Instagram, Twitter, WhatsApp); friend	3 (0.7%)	421 (99.3%)
Social media (Facebook, Instagram, Twitter, WhatsApp); mosque	13 (3.1%)	411 (96.9%)
Social media (Facebook, Instagram, Twitter, WhatsApp); newspaper	1 (0.2%)	423 (99.8%)
Social media (Facebook, Instagram, Twitter, WhatsApp); radio	3 (0.7%)	421 (99.3%)
Social media (Facebook, Instagram, Twitter, WhatsApp); radio; school	11 (2.6%)	413 (97.4%)
Social media (Facebook, Instagram, Twitter, WhatsApp); school	12 (2.8%)	412 (97.2%)
Social media (Facebook, Instagram, Twitter, WhatsApp); TV	18 (4.2%)	406 (95.8%)
Social media (Facebook, Instagram, Twitter, WhatsApp); TV; church	8 (1.9%)	416 (98.1%)
Social media (Facebook, Instagram, Twitter, WhatsApp); TV; family member	5 (1.2%)	419 (98.8%)
Social media (Facebook, Instagram, Twitter, WhatsApp); TV; friends	3 (0.7%)	421 (99.3%)
Social media (Facebook, Instagram, Twitter, WhatsApp); TV; mosque	2 (0.5%)	422 (99.5%)
Social media (Facebook, Instagram, Twitter, WhatsApp); TV; radio	24 (5.7%)	400 (94.3%)
Social media (Facebook, Instagram, Twitter, WhatsApp); TV; school teacher	7 (1.7%)	417 (98.3%)
TV	84 (19.8%)	340 (80.2%)
TV; church	4 (0.9%)	420 (99.1%)
TV; church; family member	3 (0.7%)	421 (99.3%)
TV; church; school teacher	2 (0.5%)	422 (99.5%)
TV; church; school teacher; family member	2 (0.5%)	422 (99.5%)
TV; church; school teacher; friends; family member	1 (0.2%)	423 (99.8%)
TV; family member	9 (2.1%)	415 (97.9%)
TV; friends	1 (0.2%)	423 (99.8%)
TV; friends; family member	1 (0.2%)	423 (99.8%)
TV; mosque; family member	1 (0.2%)	423 (99.8%)
TV; mosque; family member; newspaper	1 (0.2%)	423 (99.8%)
TV; mosque; school teacher; family member	1 (0.2%)	423 (99.8%)
TV; mosque; school teacher; friends; family member	1 (0.2%)	423 (99.8%)
TV; radio	10 (2.4%)	414 (97.4%)
TV; radio; church	1 (0.2%)	423 (99.8%)
TV; radio; church; school teacher; friends; family member; newspaper	1 (0.2%)	423 (99.8%)
TV; radio; family member	2 (0.5%)	422 (99.5%)
TV; radio; friends	1 (0.2%)	423 (99.8%)
TV; radio; friends; family member	1 (0.2%)	423 (99.8%)
TV; radio; mosque	2 (0.5%)	422 (99.5%)
TV; radio; school teacher	1 (0.2%)	423 (99.8%)
TV; radio; school teacher; newspaper	1 (0.2%)	423 (99.8%)
TV; school teacher	7 (1.7%)	417 (98.3%)
TV; school teacher; family member	3 (0.7%)	421 (99.3%)
TV; school teacher; friends	1 (0.2%)	423 (99.8%)
TV; school teacher; friends; family member	1 (0.2%)	423 (99.8%)
TV; school teacher; friends; family member; market	1 (0.2%)	423 (99.8%)

In Table 7, it is shown that 32.8% of the respondents will take the COVID-19 vaccine if approved, whereas 67.2% of the participants noted that they would not take the COVID-19 vaccine.

**Table 7:** Information about taking approved vaccines for COVID-19 COVID-19: coronavirus disease 2019

Item	Yes	No
Will you take the COVID-19 vaccines when they are approved for young people between 10 and 19 years of age?	139 (32.8%)	285 (67.2%)

In Table 8, it is noted that the reasons behind this refusal of vaccination are multifaceted. The leading reason was parental refusal at 57.5%, followed distantly by that it can kill at 13% and fear that they might react to it by 7%.

**Table 8:** Reasons behind refusal of vaccination (if the answer to Table 7 is "no")

Item	Number	%
Nil	4	1.4
I fear that I might react to it	20	7
It can kill	37	13
It could change my DNA	2	0.7
It is not effective	6	2.1
It is not safe	20	7
It is painful	4	1.4
It will make me unable to have children in the future	1	0.3
My imam preaches against it	10	3.5
My parents will refuse	164	57.5
My pastor preaches against it	4	1.4
The Whites are using African Americans for experiments to test the vaccine	13	4.6

In Table 9, the regression analysis for gender shows a beta value (B) of 1.477 and a P-value of 0.000, whereas that of age shows a beta value of 1.955 and a P-value of 0.000.

**Table 9:** Prevention measures for COVID-19 COVID-19: coronavirus disease 2019, SE: standard error

Predictors	SE	t-value	B	P-value
Gender	0.076	19.540	1.477	0.000
Age	0.140	13.925	1.955	0.000
R squared = 0.001 (adjusted R squared = -0.004) for gender				
R squared = 0.005 (adjusted R squared = 0.001) for age				

As seen in Table 10, a between-subjects effects test conducted showed a significance of 0.820 for gender and 0.323 for age. Furthermore, the partial eta squared values for both gender and age related to the vaccine were 0.001 and 0.005, respectively.

**Table 10:** Tests of between-subjects effects: prevention measure of COVID-19 a: R squared = 0.001 (adjusted R squared = -0.004), b: R squared = 0.005 (adjusted R squared = 0.001) COVID-19: coronavirus disease 2019

Source	Dependent variable	Type III sum of squares	df	Mean square	F	Sig.	Partial eta squared
Corrected model	Gender	0.100^a^	2	0.050	0.199	0.820	0.001
	Age	1.966^b^	2	0.983	1.134	0.323	0.005
Intercept	Gender	281.913	1	281.913	1120.956	0.000	0.727
	Age	559.777	1	559.777	645.718	0.000	0.605
COVID-19 vaccines	Gender	0.100	2	0.050	0.199	0.820	0.001
	Age	1.966	2	0.983	1.134	0.323	0.005
Error	Gender	105.879	421	0.251	-	-	-
	Age	364.968	421	0.867	-	-	-
Total	Gender	1051.000	424	-	-	-	-
	Age	2320.000	424	-	-	-	-
Corrected total	Gender	105.979	423	-	-	-	-
	Age	366.934	423	-	-	-	-

In Table 11, the parameter estimates show that the 95% confidence intervals for the vaccine variables overlap zero, and their significance values are above 0.05.

**Table 11:** Parameter estimates a: This parameter is set to zero because it is redundant. COVID-19: coronavirus disease 2019

Dependent variable	Parameter	B	Standard error	t	Sig.	95% confidence interval		Partial eta squared
						Lower bound	Upper bound	
Gender	Intercept	1.477	0.076	19.540	0.000	1.329	1.626	0.476
	(Vaccines COVID-19=1)	0.021	0.080	0.266	0.790	-0.136	0.179	0.000
	(Vaccines COVID-19=2)	-0.042	0.129	-0.329	0.742	-0.296	0.211	0.000
	(Vaccines COVID-19=3)	0^a^	-	-	-	-	-	-
Age	Intercept	1.955	0.140	13.925	0.000	1.679	2.230	0.315
	(Vaccines COVID-19=1)	0.219	0.149	1.473	0.142	-0.073	0.512	0.005
	(Vaccines COVID-19=2)	0.132	0.240	0.553	0.581	-0.338	0.603	0.001
	(Vaccines COVID-19=3)	0^a^	-	-	-	-	-	-

## Discussion

In this study, an almost equal distribution of male (50.7%) and female (49.3%) respondents was observed, enhancing the representativeness of the sample across gender lines. The age range was from 10 to 19, with the highest percentage (36.1%) falling within the 13-15 year category. The predominant religion was Christianity (76.7%), followed by Islam (20.8%), which is reflective of the broader religious composition in Nigeria [10]. The distribution of the parents' education level, both mother's and father's, is noteworthy. It is especially relevant considering that parents' education has been associated with health literacy and, consequently, health behaviors [11]. About 23.8% of the fathers and a considerably higher percentage of the mothers (39.6%) had tertiary education, potentially indicative of a better comprehension and communication of health information within the family. Nonetheless, a significant proportion of participants expressed ignorance regarding the educational attainment of their mothers (21%) and fathers (22.2%), which could potentially create challenges when evaluating the impact of parental education on the health behaviors of adolescents. It is reasonable to deduce that parents with higher education levels are more likely to have access to, understand, and pass on accurate information about the pandemic [12].

A significant finding was that all respondents (100%) were aware of COVID-19, demonstrating the broad reach of information regarding the pandemic. Various sources were cited for their awareness, including social media (46.9%), TV (31.1%), and radio (15.6%). These findings align with other studies that have highlighted the prominent role of the media in disseminating health information, especially during public health crises [13]. However, it is worth noting that sources such as social media may also proliferate misinformation, underscoring the need for authoritative bodies to monitor and regulate these platforms [14]. The minimal role of family, school, church, mosque, newspapers, and friends in raising awareness indicates a reliance on mass media over interpersonal or community-based sources.

The understanding of the causes of COVID-19 among the respondents appears to be somewhat varied. A significant number of adolescents (46.9%, n = 199) correctly identified viruses as the cause of COVID-19. However, a slightly larger proportion (52.8%, n = 224), responded negatively to this statement, revealing a misunderstanding or lack of knowledge about the cause of the disease. This is a crucial aspect, as understanding the viral nature of COVID-19 is fundamental to implementing appropriate measures to control its spread [15].

Interestingly, a considerable number of adolescents incorrectly associated bacteria (31.1%, n = 132) and fungi (15.6%, n = 66) with the cause of COVID-19. This misconstruction potentially suggests that adolescents are mixing up different types of pathogens or might have received incorrect information about the disease [16]. This demonstrates a certain level of confusion about the microbiology of infectious diseases among the respondents. It aligns with the literature, where similar misconceptions were reported in different populations globally, revealing an urgent need for targeted education campaigns [5,17].

Regarding the question about a cure for COVID-19, a majority of respondents (63.4%, n = 269) believe that the disease has a permanent cure. This reveals a significant misunderstanding, as, until the date of this study (2023), there is no specific permanent cure for COVID-19, and the primary treatments include symptom management and supportive care [18,19]. This belief in a permanent cure might be attributed to the widespread dissemination of misinformation during the pandemic [20]. However, ongoing research into antiviral medications is promising.

In terms of immunization against COVID-19, a substantial majority (84.2%, n = 357) of the adolescents were aware of the existence of vaccines, reflecting the global efforts in vaccination campaigns [21]. This is an encouraging finding, considering the pivotal role of vaccines in controlling the spread of the virus and reducing the severity of the disease [22]. It is, however, noteworthy that some adolescents (5.4%, n = 23) were unaware of the vaccines, indicating the need for continued public health education and vaccination campaigns.

A large majority of respondents (73.6%, n = 312) recognized COVID-19 as a significant issue in Nigeria and globally. This shows a generally high level of awareness about the global impact of the pandemic and aligns with the classification of the World Health Organization (WHO) of COVID-19 as a pandemic [23]. However, it is essential that the remaining adolescents who did not perceive the seriousness of the issue be reached with tailored information about the implications of the pandemic, especially in their local context [24]. This is paramount to ensure effective control and prevention of COVID-19, considering that accurate knowledge directly influences disease-related behaviors and practices [25].

The results presented in Table 4 and Table 5 show that knowledge and practice regarding the precautions for COVID-19 among the respondents were suboptimal. Remarkably, only 8.5% of the respondents acknowledged the importance of hand washing with soap and water, a basic hygiene measure recommended by health authorities worldwide as a key practice in preventing the transmission of the virus [15]. This figure is startlingly low given that hand hygiene is an elementary public health intervention that has been extensively advocated since the onset of the pandemic [26]. Avoidance of crowded places, another highly recommended precaution, was acknowledged by only 1.2% of the respondents. This is concerning, as avoidance of large gatherings and adherence to social distancing have been shown to significantly reduce the spread of the virus [27]. Such a low figure may suggest that public health messages may not have adequately reached or resonated with this demographic. The rate of adoption of COVID-19 vaccination was also alarmingly low, at just 1.2%. This might suggest a lack of accessibility to the vaccine, hesitancy, or misinformation among the studied population, all of which can have severe implications for the control of the virus' spread [28]. This finding is similar to a study by Olapegba et al. [29], which found that hand washing and social distancing were the most recognized precautions against COVID-19 in Nigeria. The regular use of hand sanitizer was also not widely practiced among the respondents, with only 0.7% indicating its usage. Hand sanitizers, especially those with at least 60% alcohol, are deemed effective in killing many types of germs, including the coronavirus, when soap and water are not readily available [30]. The most adopted combination of precautions was hand washing with soap and water, social distancing, and the regular use of hand sanitizer, yet this was still only at a rate of 27.4%. This is an alarming statistic, considering these combined methods are considered some of the most effective strategies in preventing the transmission of the virus [31]. Similarly, 51 (12%) respondents recognized the combination of hand washing with soap and water, regular use of hand sanitizer, and COVID-19 vaccination. This finding is in line with research conducted by Umakanthan et al. [32], which emphasized how preventive strategies play a major role in reducing the public spread of the virus.

Despite these recognitions, the overall level of knowledge among respondents is significantly low considering that more than 90% of the respondents did not recognize essential precautions such as vaccination, social distancing, and avoiding crowded places. This result aligns with previous studies in low- and middle-income countries, including Nigeria, where the level of knowledge and practice of COVID-19 preventive measures was found to be significantly low [33]. Interestingly, the study also found that some respondents recognized non-scientifically proven precautions such as special prayers, regularly drinking hot water, eating bitter kola/pepperfruit, and bathing with salt water. These findings show misinformation, superstitious beliefs, and myths prevalent among the respondents, which can significantly impede effective prevention and control of the disease [14,34]. This indicates a need for further education and information dissemination [35].

The results of this study imply an alarming deficit in the implementation of proper and recommended safety measures against COVID-19 among the adolescent population in Umuahia, Abia State. The most common precautionary practice, as reported by the respondents, is hand washing with soap and water. A total of 53 (12.5%) respondents reported adhering to this essential safety practice, reflecting the emphasis put on hand hygiene during the pandemic by the World Health Organization (WHO) and the Nigeria Centre for Disease Control (NCDC) [15,36]. This finding corresponds with the global trend observed in numerous studies, highlighting the importance and effectiveness of hand hygiene in controlling the spread of COVID-19 [37,38]. The result also reveals that 81 (19.1%) respondents claimed that they practiced social distancing in conjunction with hand washing. This suggests some understanding of the combined effect of these measures in controlling the spread of the virus. However, this rate is still disturbingly low given the proven effectiveness of these combined measures in disease prevention [27].

It is disconcerting that the third most prevalent measure (the use of hand sanitizer along with hand washing and social distancing) was reported by only 44 (10.4%) respondents. It suggests a relatively low rate of adherence to this highly recommended precaution, despite the well-established antimicrobial efficacy of hand sanitizer against the SARS-CoV-2 virus [30]. A discouragingly low number of participants reported adhering to other critical precautions such as using face masks and avoiding crowded places, both less than 1%. This is alarming, considering that using masks and avoiding crowded places are critical factors in the prevention of COVID-19 transmission [39]. The results also indicate that some adolescents are engaging in practices with no proven effectiveness against COVID-19, such as eating bitter kola/pepperfruit or drinking hot water. This highlights the misinformation surrounding COVID-19 and the urgent need for public health education interventions targeting adolescents.

The vaccination rate among respondents is also alarmingly low (less than 1%). This could be due to a variety of reasons such as vaccine accessibility, vaccine hesitancy, or misinformation about vaccines [40]. The government and public health officials should address these issues to increase the uptake of COVID-19 vaccinations among adolescents. Analysis of the data indicates that the most prominent source of knowledge about the COVID-19 vaccines among respondents is television (19.8%), followed by radio (10.6%). Social media also emerges as a key source of information, with 8.3% of respondents citing platforms such as Facebook, Instagram, Twitter, and WhatsApp. This mirrors global trends, wherein social media has become a central information source, especially among younger demographics [14]. This aligns with the idea that media channels continue to play a pivotal role in health education, especially in areas where health literacy is low, and there is a need for accessible and straightforward information dissemination [41]. Moreover, the rising influence of social media platforms in disseminating health information to adolescents, as found in other studies [42], is consistent with our results.

However, the data also points to an alarming trend: the majority of adolescents, irrespective of the information source, still have insufficient knowledge about the sources of COVID-19 vaccines. This is evident from the large percentage (ranging from 80.2% to 99.8%) of respondents answering "no" to various knowledge sources. This gap in knowledge could potentially expose this demographic to misinformation and lead to vaccine hesitancy, a significant public health issue amid a pandemic [43]. It is noteworthy that traditional community structures such as churches and mosques seem to have a minimal role in providing vaccine-related information. In African communities, religious organizations often hold a key position in social and community life, but their role in disseminating knowledge about the COVID-19 vaccines appears minimal based on the data [44]. This highlights a potential area where public health campaigns could collaborate with these religious organizations to effectively reach adolescents. Another key point from the data is the underutilization of schools as a source of information. Only a few respondents identified school teachers as a source of information. School-based health education programs have been found to be effective in improving health knowledge among adolescents, suggesting that there is a missed opportunity in this area [45].

As can be observed from the results (Table 7), there is a considerable level of vaccine hesitancy among the studied population, with several factors influencing their decision not to take the vaccine. The results reveal that a significant majority (67.2%) of the participants would not take the COVID-19 vaccines when approved for their age group. The reasons behind this resistance to vaccination are multifaceted, as shown in Table 8. The most prominent reason for refusal among the respondents is parental refusal, accounting for 57.5% of negative responses. This aligns with the findings of some earlier studies that suggest that parental attitudes play a crucial role in adolescent vaccination decisions [46]. Additionally, the adolescents' reasons for not wanting to take the vaccine ranged from fear of side effects (e.g., allergic reactions, pain, and potential harm to future reproductive capabilities) to questioning the vaccine's safety and efficacy. These fears have been recognized in various studies, with perceived risks influencing vaccine hesitancy [47]. In particular, the fear of the vaccine changing one's DNA, although scientifically unfounded, shows a misunderstanding of how mRNA vaccines work, suggesting a need for clear, accurate information to address these misconceptions [18]. These findings echo the results of other studies on vaccine hesitancy, which highlight concerns about safety and side effects as recurrent themes among those reluctant to get vaccinated [28].

Concerns about vaccines being part of a larger conspiracy were also noted, including the claim of vaccines being used for experimentation on specific racial groups. This mirrors historical vaccine hesitancy stemming from racial disparities and mistrust in medical establishments [48]. A percentage of participants (13%) expressed the fear that the vaccine could cause death, while 2.1% doubted its effectiveness. Misconceptions about the vaccine were also prevalent, with 0.7% believing that it could change their DNA and 4.6% endorsing the conspiracy theory that the vaccine is an experiment on African American people. These beliefs have been debunked by the scientific community [49,50], but their presence in the population's belief system indicates a need for better public health communication strategies. Religion also plays a part in vaccine hesitancy, with 3.5% of participants stating that their imam preaches against it and 1.4% indicating that their pastor preaches against it. This suggests that religious leaders can significantly influence health behaviors, which is consistent with previous research [51,52].

In the regression analysis as shown in Table 9, the results indicate that both gender and age are significant predictors for the prevention measures of COVID-19. The beta value (B) of 1.477 and a P-value of 0.000 for gender suggest a strong positive relationship with COVID-19 prevention measures. Similarly, for age, the beta value of 1.955 and a P-value of 0.000 indicate a strong positive association with COVID-19 preventive measures. However, it is worth noting that both predictors accounted for a very small proportion of variance in the dependent variable. The adjusted R-squared values for gender and age were -0.004 and 0.001, respectively, which points to the limited contribution of these predictors to the overall model [53]. The low R-squared value implies that these predictors, gender and age, account for a very small portion of the variance in prevention measures [54].

In Table 10, a between-subjects effects test was conducted. This test revealed that there is no significant relationship between the COVID-19 vaccine and the dependent variables, gender and age. The significance values (0.820 for gender and 0.323 for age) are well above the threshold of 0.05, suggesting that the results may be due to chance. This implies that the COVID-19 vaccine does not significantly contribute to explaining the variance in gender and age for the practice of COVID-19 prevention measures. Similarly, the partial eta squared values for both gender and age related to the vaccine were very low (0.001 and 0.005, respectively), pointing to the limited effect size of the vaccine on these variables [55].

Finally, in the parameter estimates (Table 11), it is apparent that neither the gender nor the age groups who have received various levels of the COVID-19 vaccine (represented by the Vaccines COVID19 variables) show a significant influence on COVID-19 prevention measures. The 95% confidence intervals for all the vaccine variables overlap zero, and their significance values are above 0.05. This reinforces the notion that the COVID-19 vaccine does not significantly affect the practice of COVID-19 prevention measures among adolescents in Umuahia, Abia State, Nigeria.

Recommendations

Based on the findings of this study, the following recommendations can be made.

Improvement of Public Health Education

It appears that the majority of respondents lack comprehensive knowledge about the causes of COVID-19 and the appropriate precautionary measures to take. As such, public health education should be enhanced and tailored to adolescents' comprehension. The curriculum should focus on basic virus transmission knowledge and standard prevention measures such as proper hand hygiene, social distancing, and wearing masks. Also, adequate preparation of physicians with the needs of the current time is important to address the situations that patients are having.

More Effective Use of Media Platforms

The primary source of COVID-19 awareness among respondents is social media and television. Thus, these platforms should be more effectively utilized to disseminate accurate information about COVID-19. This could involve engaging popular influencers to deliver important public health messages or producing more engaging, teen-friendly content about COVID-19 prevention and control.

Targeted Vaccination Awareness

While a significant number of respondents were aware of the existence of COVID-19 vaccines, not many had taken them or knew the importance of the precautionary measures. Therefore, awareness campaigns should not only emphasize the availability of vaccines but also their safety, efficacy, and the critical role they play in preventing disease transmission and severity.

Involvement of Parents in Health Education

Given that a significant number of parents had tertiary education (based on the tables), they could be engaged as critical stakeholders in improving the adolescents' knowledge of COVID-19. By providing parents with correct and up-to-date information, they can reinforce health messages and practices at home.

Addressing Misinformation

Some adolescents held misconceptions about the virus' causes and ineffective prevention measures (such as bathing with salt water or drinking concoctions). Future public health efforts should also address these misconceptions by providing accurate information and debunking common myths about the virus.

School-Based Health Education Programs

School-based health education programs could be beneficial, considering that many adolescents attend school. These programs can ensure the consistent and accurate delivery of health messages to this target group.

Monitoring and Evaluation

Continuous assessment of knowledge, attitudes, and practices regarding COVID-19 among adolescents in the region is crucial to inform and adjust strategies and interventions effectively. Regular surveys and studies, similar to this one, will be important for monitoring changes over time.

Limitations

Despite our efforts to give a reliable and detailed examination of our study objectives, our evaluation has limitations. As with all cross-sectional studies, this study design limits our ability to establish causality. When relying on self-reported information, there is a risk of recall bias, where participants may not accurately remember or report their knowledge and practice of the precautions and preventions they take.

The knowledge and practice of precautions and prevention of COVID-19 can change rapidly due to factors such as ongoing research studies, public health interventions, and changes in population behavior. A cross-sectional study captures data from only one point in time, which might not reflect ongoing trends or fluctuations. In the future, increasing the sample size for the power of analysis will be helpful to have more generalizable results [56].

## Conclusions

The results indicate a varied level of understanding about COVID-19 among adolescents in Umuahia, Abia State, Nigeria. While the majority have a good grasp of some aspects, there are still significant misconceptions and knowledge gaps that need to be addressed, particularly regarding the cause and cure of the virus. The study indicates that while gender and age may be statistically significant predictors for the practice of COVID-19 prevention measures, they account for a very small proportion of the variance.

Furthermore, receiving the COVID-19 vaccine does not significantly influence these practices among the adolescent population studied. These findings call for intensified public health education to improve the level of knowledge and practice of COVID-19 precautions among this demographic, with a particular focus on debunking myths and misinformation regarding the disease.
